# First Insight into the Genome Sequence of Clostridium liquoris DSM 100320, a Butyrate- and Ethanol-Producing Bacterium

**DOI:** 10.1128/genomeA.00376-18

**Published:** 2018-05-03

**Authors:** Anja Poehlein, Hannes Neubauer, Philipp Niemeyer, Rolf Daniel

**Affiliations:** a Genomic and Applied Microbiology & Göttingen Genomics Laboratory, Institute of Microbiology and Genetics, Georg-August University of Göttingen, Göttingen, Germany; b Members of the Applied Bioinformatics in Microbiology Course of the Microbiology and Biochemistry MSc/PhD Program, Georg-August University of Göttingen, Göttingen, Germany

## Abstract

Clostridium liquoris is a strictly anaerobic, Gram-positive, nonmotile, spore-forming, rod-shaped bacterium. The major fermentation products from glucose are ethanol and butyrate. C. liquoris was isolated from a 20-year-old liquor fermentation pit. The draft genome sequence consists of a chromosome (2.892 Mb) harboring 2,788 predicted protein-encoding genes.

## GENOME ANNOUNCEMENT

Clostridium liquoris DSM 100320 is a strictly anaerobic, Gram-positive, nonmotile, spore-forming, rod-shaped bacterium. It was isolated from the mud of a 20-year-old fermentation pit used for production of Chinese strong-flavored liquor in Mianzhu, Sichuan, China (1). Optimal growth conditions are 37°C and a pH value between 7.5 and 8.5 (1). The major fermentation products from glucose are ethanol and butyrate. In contrast to its closest phylogenetic neighbors, C. lundense and C. tetanomorphum, C. liquoris can utilize a plethora of substrates as carbon sources, including maltose, mannitol, trehalose, lactose, mannose, glycerol, xylose, and cellobiose (1).

The DNA from C. liquoris was isolated by using the MasterPure complete DNA purification kit according to the protocol of the manufacturer (Epicentre, Madison, WI). Illumina paired-end sequencing libraries were generated by using the recovered DNA as recommended by the manufacturer (Illumina, San Diego, CA). A MiSeq instrument and MiSeq reagent kit version 3 were employed to sequence the generated sequencing libraries, as recommended by the manufacturer (Illumina, San Diego, CA). Quality trimming using Trimmomatic version 0.36 (2) resulted in 2,245,314 paired-end reads. Assembly carried out with SPAdes version 3.11.1 (3) yielded 75 contigs (>500 bp), with a 160-fold average coverage. Validation of the assembled contigs was achieved by employing Qualimap version 2.2.1 (4). Automatic gene prediction was executed with the software tool Prokka (5). The draft genome sequence consists of a chromosome (2.892 Mb) with a GC content of 30.96% and 2,788 predicted protein-encoding genes, of which 2,065 had predicted functions. Furthermore, 60 tRNA, 8 rRNA, and 1 transfer-messenger RNA (tmRNA) genes and 3 repeat regions were identified. Twelve putative clustered regularly interspaced short palindromic repeat (CRISPR)/Cas-related and 25 phage-associated genes were identified. The PHAge Search Tool Enhanced Release (PHASTER) software tool (6, 7) predicted 3 prophage regions. Moreover, 13 putative genes encoding multidrug resistance proteins, including 5 multidrug exporters, were identified.

Ethanol and butyrate are the major fermentation products of C. liquoris and are accompanied by smaller amounts of lactose and acetate (1). Correspondingly, genes associated with the production of these organic acids, such as putative genes encoding 3-hydroxybutyryl-coenzyme A (CoA) dehydrogenase, enoyl-CoA hydratase, acyl-CoA dehydrogenase, succinyl-CoA:coenzyme A transferase, and butyrate kinase, which were located in the vicinity of a phosphate butyryltransferase-encoding gene, were found in the genome. Additionally, genes encoding an l-lactate dehydrogenase, an acetate kinase (*ack*), and a phosphate acetyltransferase (*pta*) were identified, whereby *ack* and *pta* form an operon-like structure. Putative genes involved in solvent production, such as genes encoding an alcohol dehydrogenase and NADH-dependent butanol dehydrogenase, were also present.

### Accession number(s).

This whole-genome shotgun project has been deposited in DDBJ/EMBL/GenBank under the accession number PVXO00000000. The version described here is the first version, PVXO01000000.
